# Three-dimensional anatomy of the Denonvilliers’ fascia after micro-CT reconstruction

**DOI:** 10.1038/s41598-021-01106-8

**Published:** 2021-11-05

**Authors:** Seung Yoon Yang, Ho Seung Kim, Min Soo Cho, Nam Kyu Kim

**Affiliations:** Division of Colon and Rectal Surgery, Department of Surgery, Severance Hospital, Yonsei University College of Medicine, 50-1 Yonsei-ro, Seodaemun-Gu, Seoul, 03722 Korea

**Keywords:** Cancer, Anatomy, Gastroenterology

## Abstract

An understanding of the anatomy of the Denonvilliers’ fascia is essential for successful surgical outcomes for patients with rectal cancer in the mid- to lower regions, especially near the seminal vesicles and prostate in males. Whether the correct surgical plane during a total mesorectal excision should be anterior or posterior to the Denonvilliers’ fascia is currently under debate. This study aimed to investigate the Denonvilliers’ fascia using micro-computed tomography (micro-CT) to acquire three-dimensional images nondestructively for assessments of the relationship between the Denonvilliers’ fascia, the mesorectal fascia, and neurovascular bundles to elucidate the correct anterior total mesorectal excision plane. Eight specimens were obtained bilaterally from four fresh human cadavers. Four specimens were stained with phosphotungstic acid to visualize the soft tissue, and micro-CT images were obtained; the other four specimens were stained with Masson’s trichrome to visualize connective tissue. Micro-CT images corroborate that the Denonvilliers’ fascia consists of a multilayered structure that separates the rectum from the seminal vesicles and the prostate. Specimens stained with Masson’s trichrome showed that the urogenital neurovascular bundle located at the posterolateral corner of the prostate is separated from the mesorectum by the Denonvilliers’ fascia. For the preservation of autonomic nerves necessary for urogenital function and optimal oncologic outcomes in patients with rectal cancer, a successful mesorectal excision requires a dissection plane posterior to the Denonvilliers’ fascia.

## Introduction

The structural boundaries between the Denonvilliers’ fascia (DVF) and the mesorectal fascia (MRF), a layer of connective tissue enclosing the mesorectum which forms the circumferential resection margin, in patients with rectal cancer remain controversial and appear to be related to the appropriate surgical plane during anterior total mesorectal excision (TME). Identifying the DVF is a critical step in TME, enabling a surgeon to mobilize the rectum safely. Performing a dissection anterior to the DVF may increase the risk of urogenital dysfunction due to nerve damage, including that of the neurovascular bundles (NVBs)^1–4^. However, while executing a dissection posterior to the DVF can minimize the risk of nerve injury, this approach increases the positivity of circumferential resection margins in anteriorly located tumors^5–7^.

Originally, Heald et al. described that a dissection performed anterior to the DVF is an oncologically safe TME plane because it constitutes the anterior surface of the mesorectum^8,9^. Further, these authors stressed that to prevent injury to the NVBs, it is necessary to identify and preserve both edges of the DVF using a U-shaped incision^9^. This interpretation of the DVF is consistent with those of previous studies in which the origin of the DVF is described as a fused, single layer of two primitive fetal peritoneal layers that pass between the rectum posteriorly and the prostate or seminal vesicles anteriorly^2,10,11^. However, this concept of fused embryonic layers has been challenged by some who insist that the DVF is actually the compression of multiple layers of mesenchymal tissue^5,12^.

Based on this “multilayer theory,” some studies have reported that dissecting the posterior region of the DVF prevents damage to the NVBs during TME, which subsequently reduces postoperative urogenital dysfunction. Therefore, the recommended optimal plane for TME is anterior to the MRF and posterior to the DVF unless the tumor is locally advanced or anteriorly located^6,7^. Nevertheless, the in situ three-dimensional structures of the DVF have not been previously described because their topography and relationship to the surrounding soft tissues are very complex^7,13^. In this study, we used micro-computed tomography (micro-CT) with the staining of soft tissues to observe the three-dimensional structures of the DVF and its surrounding environment in detail^14,15^.

Therefore, the aim of this study was to clarify the three-dimensional structures of the DVF and their association with the MRF using micro-CT and to verify the anatomic relationship between the DVF and NVBs using histological analyses.

## Materials and methods

### Specimens

Four whole pelvises, extending from the fourth lumbar vertebra to the upper thigh, from four fresh male cadavers (mean age 77.0 years, range 73–81 years) were studied. All specimens from individuals with a history of a pelvic tumor, severe inflammatory pelvic disorder, or major pelvic procedure that could alter the normal anatomical structure were excluded. Eight hemipelvis were obtained bilaterally from four unembalmed male cadavers. Specimens including the rectum, DVF, urinary bladder, seminal vesicles, and prostate were harvested and immediately fixed in 10% formalin for 2–3 days. Four specimens were stained with phosphotungstic acid (PTA) to visualize the soft tissue, and micro-CT images were obtained. The other four specimens were stained with Masson’s trichrome to visualize connective tissue. The specimens were divided into their medial, central, and lateral regions. All cadavers utilized in this study were legally donated to the Surgical Anatomy Education Centre at the Yonsei University College of Medicine. The Ethics Committee of the Yonsei University College of Medicine, approved this study for the given purpose and Informed consent was obtained from a next of kin and/ or legal guardian of the cadaver. All methods were carried out in accordance with relevant guidelines and regulations.

### Micro-CT images and three-dimensional image analyses

The specimens were placed in a graded series of 30%, 50%, and 70% ethanol for serial dehydration and then stained in a 1% PTA/70% ethanol solution for 5–7 days. The specimens were then scanned (Skyscan 1173, Bruker, Kontich, Belgium) using an image pixel size of 20 μm with 2240 × 2240 pixels. An electron-accelerating voltage of 70 kV and a current of 114 μA were used. The detector was set to 500 ms, and a 0.3-degree rotation step was employed. After scanning, we used NRecon software (Bruker) to convert CT data into image files that could handle on a computer. Ring Artifacts Reduction of 7 and Beam-hardening Correction of 40 were the parameters used for this study. Converted serial sectional images were observed with 3D volume-rendering software. All samples were observed using CTvox (version 2.7, Bruker), a concise but powerful software for visualizing practical volume-rendered images. Once the converted dataset had loaded, 3D analysis of internal structures was done by simply Moving and Rotating the plane of a Clipping Box and turning on the Lighting option to provide more realistic images^15^.

### Histological analyses

The four specimens from eight hemipelvis were stained with Masson’s trichrome to visualize connective tissue. The specimens were divided into medial, central, and lateral regions and further sectioned into horizontal and sagittal.

Masson’s trichrome stain was used to distinguish connective tissue, muscle tissue, and collagen fibers. The fixed specimens were embedded in paraffin, cut into 5-μm-thick sections, every 10th section was collected and mounted onto silane-coated glass slides. The sections were preserved in Bouin fluid (60 °C) for one hour prior to the application of Weigert’s iron hematoxylin. The sections were soaked in a Biebrich scarlet/acid fuchsin solution and then differentiated in a phosphomolybdic acid/PTA solution. After the sections were incubated in an aniline blue solution, 1% acetic acid was applied. The sections were then dehydrated using ethanol and cleared in xylene. Finally, the sections were evaluated using an optical microscope (BX51; Olympus, Inc., Tokyo, Japan).

## Results

**Figure 1:**
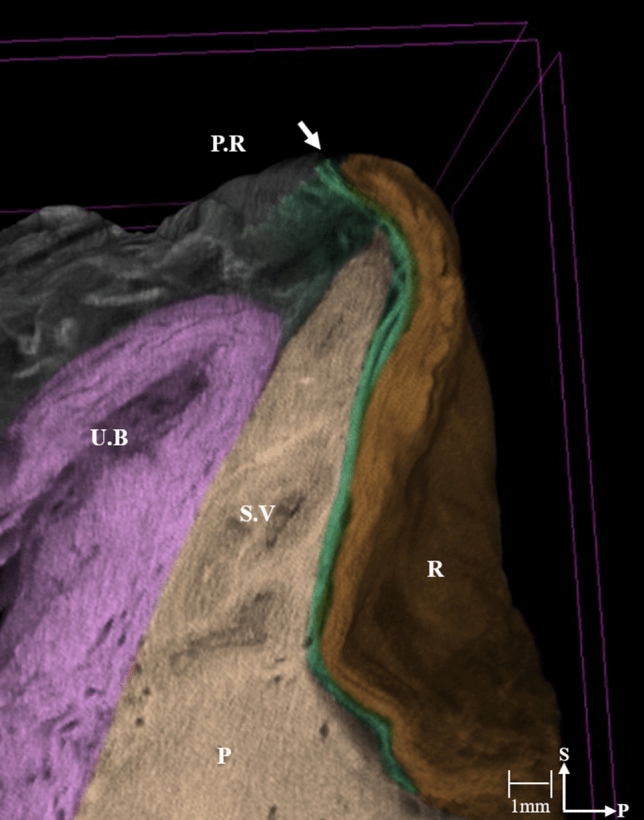
Three-dimensional anatomy of the hemi-pelvis reconstructed from micro-computed tomographic images. The arrow in in the micro-computed tomographic image indicates the deepest point of the rectovesical pouch where the DVF extends. (DVF; green). The 3D morphology of the DVF is also shown as a movie in Supplemental Video 1. PR, peritoneal reflection; UB, urinary bladder; SV, seminal vesicle; P, prostate; R, rectum; S, sagittal; P, posterior.

**Figure 2:**
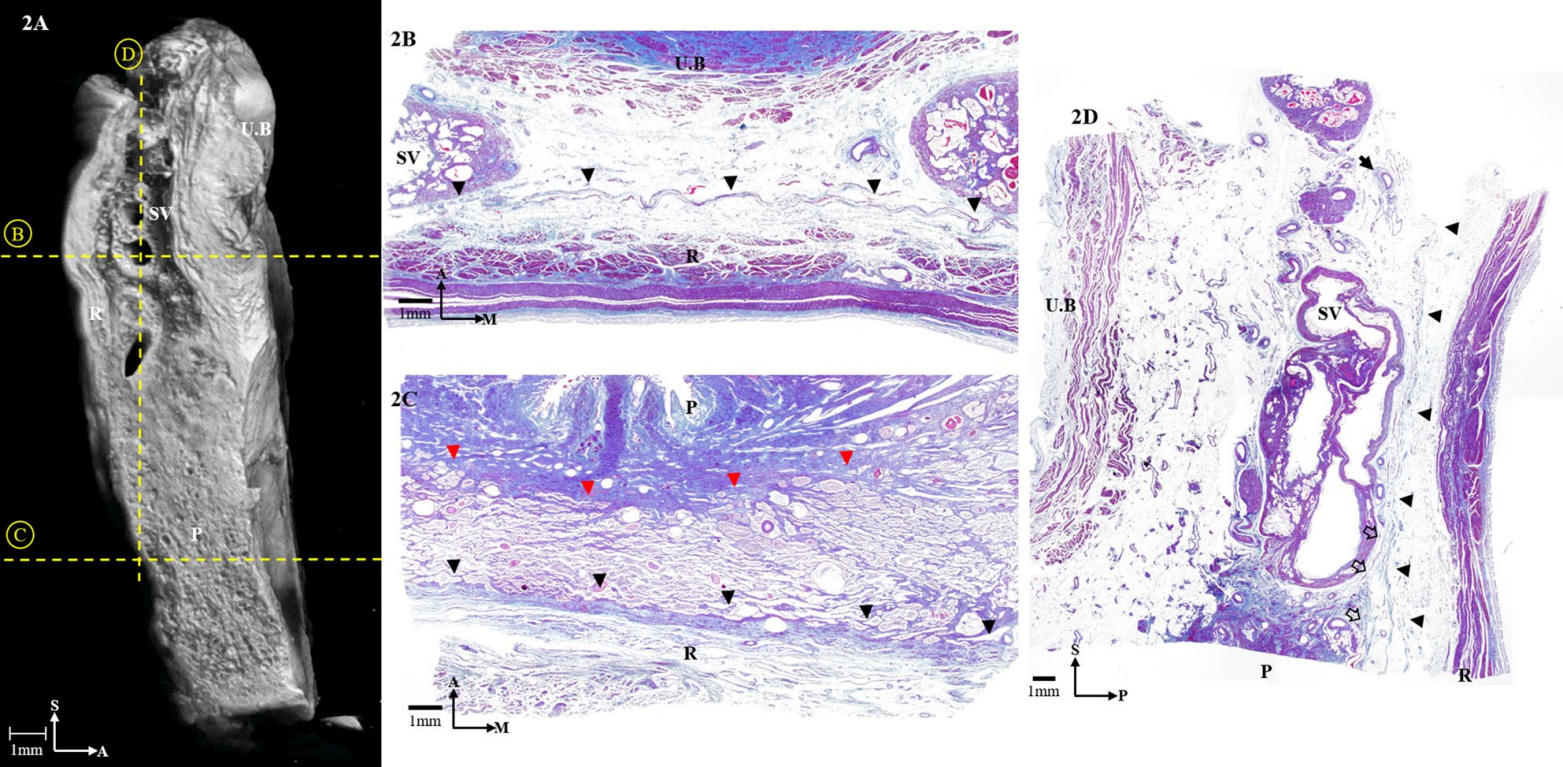
(**A**) Three-dimensional anatomy of the hemi-pelvis from a sagittal section reconstructed from micro-computed tomography. (**B**) Masson trichrome-stained section at the level of the seminal vesicles. Horizontal sections of the Denonvilliers’ fascia (DVF) are indicated by the black arrowheads. (**C**) Masson trichrome-stained section at the level of the prostate. Horizontal sections of the DVF are indicated by the black arrowheads and of the prostatic capsule is indicated by the red arrowheads. (**D**) Masson trichrome-stained section of the specimen. The black arrowheads indicate sagittal sections of the DVF, and the black arrow indicates the neurovascular bundle. The distal seminar part of the DVF merging with the prostatic capsule is indicated by the white arrows. UB, urinary bladder; SV, seminal vesicle; P, prostate; R, rectum; S, sagittal; M, medial; A, anterior; P, posterior.

The micro-CT images revealed a continuous thin plate corresponding to the DVF, located posterior to the urinary bladder, seminal vesicles, and prostate. The wide lateral portion was continuous with the lateral pelvic fascia anteriorly, covering the prostate, and, together with the MRF, surrounded the rectum and mesorectum posteriorly. Multiple laminae of the medial DVF appeared fused to the MRF, with only the inferolateral region of the DVF able to be distinguished from the MRF (Fig. 1, Supplemental Video 1). The histologic findings showed that the DVF consisted of a multilaminar structure of condensed medial and pan-shaped dispersed lateral portions as well as a mixture of collagenous, connective, and elastic tissues (Fig. 2B–D).

The DVF was located caudally to the peritoneum’s deepest point at the rectovesical pouch. A thin, multilayered structure was visualized along the superior surface of the urinary bladder without any attachment to the peritoneum (Fig. 1). Starting from the posterolateral corner of the seminal vesicles and prostate, the DVF appeared as a homogenous monolayer that was adherent to the medial prostate in both the coronal and sagittal sections. Its inverse trapezoidal shape was continuous with the perineum. At the level of the prostate, the DVF was easily distinguished from the MRF to the perineal body (Supplement Video 1).

At the level of the seminar vesicles, the micro-CT images showed that the DVF separated the seminal vesicles from the rectum (Fig. 2A). The lateral edge of the DVF appeared as a pan-shaped, multilayered structure with intervening spaces that harbored abundant adipose tissue and tiny NVBs. The number of NVBs was low in the medial region; most were located between the DVF and the urinary bladder or seminal vesicles.

**Figure 3:**
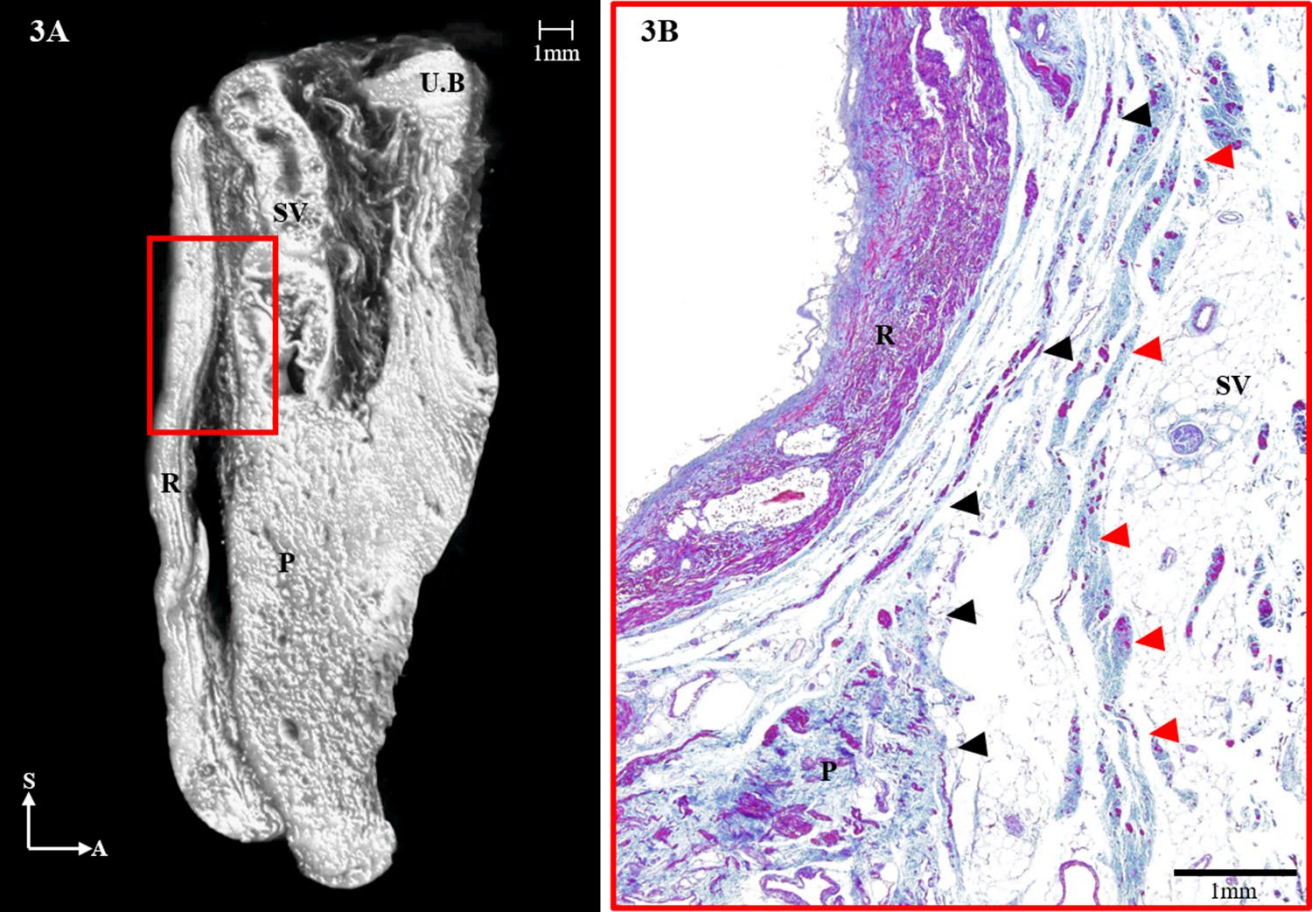
(**A**) Three-dimensional anatomy of the Denonvilliers’ fascia (DVF; red) from a sagittal section reconstructed from micro-computed tomography. (**B**) The distal, seminal part of the DVF is shown diverging to the anterior sheet and merging with the prostatic capsule (red arrowheads) and the posterior sheet (black arrowheads), covering the rectum. UB, urinary bladder; SV, seminal vesicle; P, prostate; R, rectum; S, sagittal; A, anterior.

Histologically, the DVF was observed as a multilaminar, fibromuscular tissue, whose medial region appeared condensed and seemed to widen laterally. The innermost part of the laminae contained the MRF; however, it was intricately condensed and thus indistinguishable from the DVF (Fig. 2B–D). The lowermost region of the DVF was divided into an anterior sheet that was continuous with the prostatic capsule and a posterior sheet (mesorectal fascia) that covered the rectum (Fig. 3A,B). In the micro-CT images, the lateral portion of the DVF distinctly separated the two sheets, but the medial portion of the DVF appeared condensed within a narrow area between the rectum and the prostate (Fig. 3A). The histologic study also revealed the separation of the DVF in which the anterior sheet curved anteriorly and covered the seminal vesicles while the posterior sheet enveloped the mesorectum (Fig. 3B).

**Figure 4:**
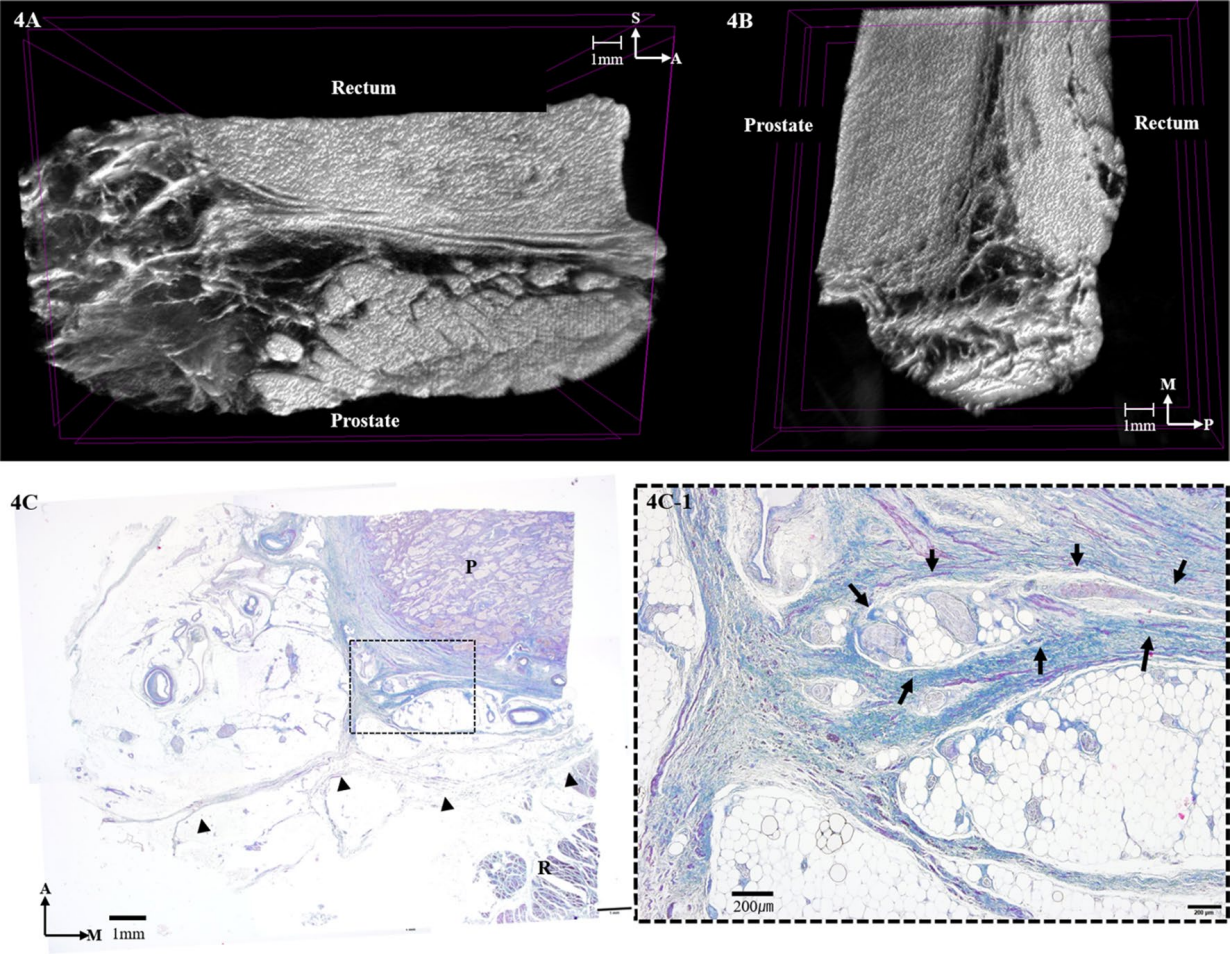
Three-dimensional anatomy of the Denonvilliers’ fascia (DVF) at the level of the prostate reconstructed from micro-computed tomography. (**A**) Horizontal section. (**B**) Sagittal section. (**C**) Masson trichrome-stained section at the level of the prostate. Horizontal sections of the DVF are indicated by the black arrowheads. Detail window C-1 shows that DVF is as closely related to the prostatic capsule and the neurovascular bundles, which are indicated by the black arrows. P, prostate; R, rectum; M, medial; A, anterior; P, posterior.

At the level of the prostate, the condensation of the medial region and the thinning of the lateral region of the fasciae appeared similar to the seminal region of the DVF (Fig. 4A,B). Continuous sagittal micro-CT images showed that the DVF demarcated the prostate, the rectum, and the NVB located between the DVF and the prostate (Fig. 4A). On the other hand, the DVF covered the prostate and joined the mesorectum, which has very rarely been observed. The histologic results revealed that the fascia was very near the prostate with the NVB situated between them (Fig. 4C). As the space between the rectum and prostate narrowed, the DVF appeared as a condensed fibrous plate that was firmly attached to the prostatic fascia.

## Discussion and conclusion

In the present study, images obtained using micro-CT and Masson’s trichrome staining showed that the DVF consists of a multilayered structure containing a mixture of collagenous, connective, and elastic tissues with smooth muscle fibers separating the rectum from the seminal vesicles and the prostate. Moreover, the urogenital NVB at the posterolateral corner of the prostate was separated from the mesorectum.

The DVF is an essential surgical landmark because, regardless of the direction of the dissection, it is associated with urogenital dysfunction as well as oncologic outcomes during TME, a standard surgery for patients with rectal cancer. It is crucial to understand the anatomy of this complex and thin structure accurately because it is challenging to identify the DVF in clinical practice, even if surgery is performed laparoscopically or robotically, which commonly provides a better surgical view.

TME respects the embryonic plane and involves an en-bloc resection of the tumor and its surrounding mesorectum via a sharp dissection of the visceral plane from the parietal fascia to secure oncologic safety^16,17^. However, in contrast to improvements in oncologic outcomes, the incidence of urogenital dysfunction remains high due to pelvic autonomic nerve (PAN) injury during surgery^17–19^. Thus, the relationship between the DVF, the MRF, and the NVBs has been studied, particularly in male patients with rectal cancer. Much controversy in the literature concerns the origin and development of the fascia, which has been described as either fused embryonic layers or compressed multiple layers of mesenchymal tissue to form a multilayered structure^10^. Recent studies have described the DVF as a multilayered, membranous, fascial structure instead of a single, membranous layer located behind the prostate and between the seminal vesicles and the rectum that developed from the fusion of the embryonic peritoneum of the rectovesical cul-de-sac, the original description of Charles-Pierre Denonvilliers^1,2,5,7,12^.

In the present study, the micro-CT images reveal the DVF as a continuous thin plate with multiple layers of condensed medial and pan-shaped dispersed lateral portions. The DVF can be distinguished from the MRF in its lateral portion, but multiple layers appear compressed with the MRF, with the two fasciae indistinguishable in the medial region. These features are commonly observed in the peritoneal reflection, at the level of the seminal vesicles, including the fusion of the DVF with the prostatic fascia. These findings support those of previous studies describing the DVF as a multilayered, membrane-like structure with the DVF and the MRF as distinctive layers, indicative of an obvious surgical plane between them^1,2,5,7,12^. Furthermore, these findings provide evidence that the posterior DVF should be an adequate dissection plane during the performance of an anterior TME without violating the key principle that the MRF should be preserved intact unless the tumor is locally advanced or anteriorly located.

**Figure 5:**
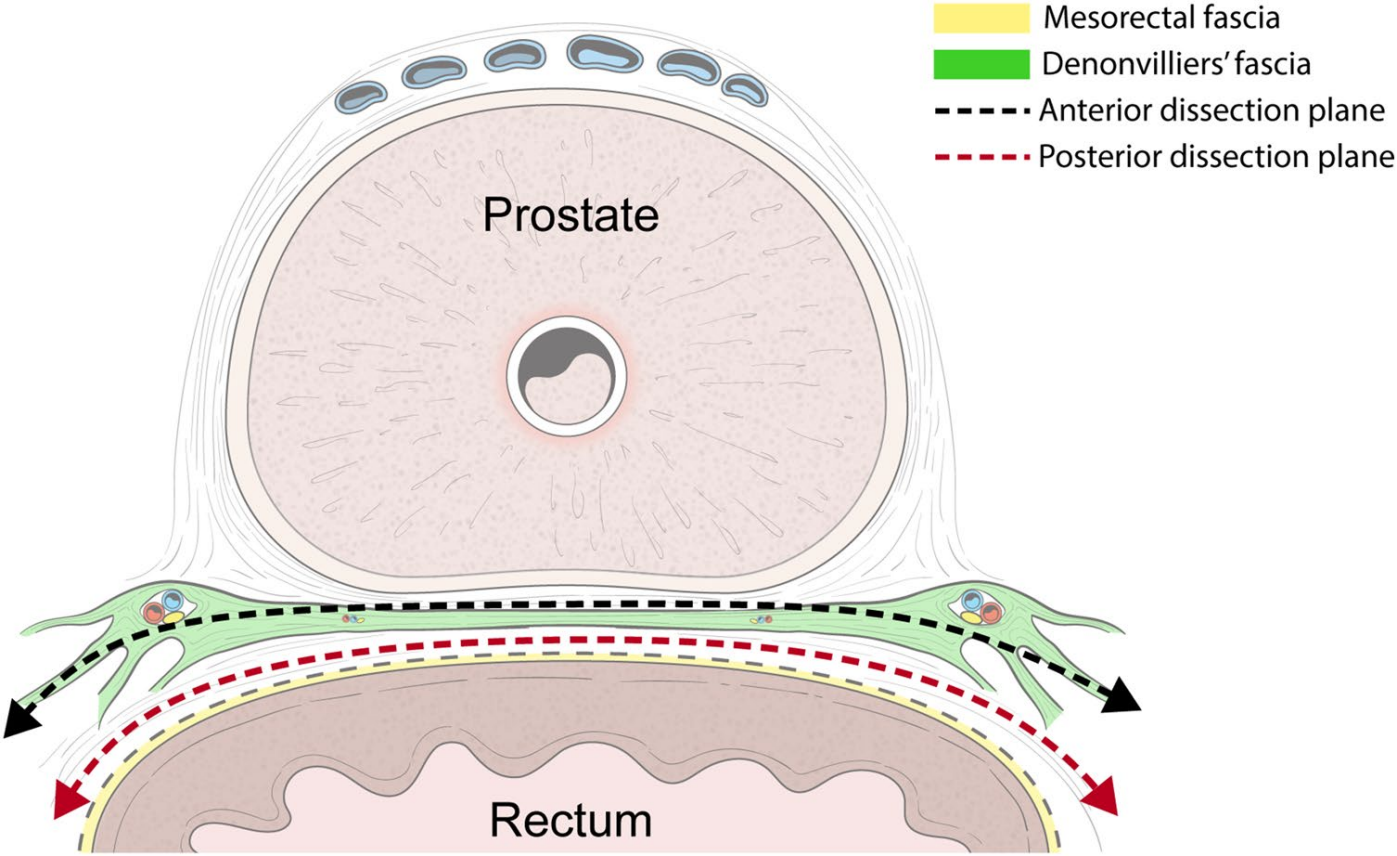
Schematic representation of the relationships of the correct surgical plane during an anterior total mesorectal excision to the Denonvilliers’s fascia in the male. The authors would like to thank Dong-Su Jang, MID (Medical Illustration & Design), a part of the Medical Research Support Services of sei University College of Medicine, for all artistic support related to this work.

Originally, Heald et al. reported that the correct TME plane should be anterior to the DVF to secure a safe, circumferential resection margin, as the DVF is part of the mesorectum’s anterior surface; these authors stressed that the surgeon should identify and preserve both edges of the DVF with a U-shaped incision to avoid postoperative urogenital dysfunction^8,9^. With Masson’s trichrome stain, we confirmed that the PAN was located between the DVF and the seminal vesicles and prostate even though the distribution of the PAN decreased in the medial region of the DVF. It is well known that the PAN extends immediately in front of the DVF to communicate with the bilateral pelvic plexuses; thus, using a surgical plane anterior to the DVF may cause erectile dysfunction in males^20^. Our micro-CT images and histologic findings are consistent with the results of previous studies in that at the prostate level, the lateral continuation of the DVF separates the urogenital NVB from the MRF^1,11^. Furthermore, we observed that the multiple layers of the DVF divide the posterior sheet covering the rectum, while the anterior sheet forms a tight fibrous plate that is firmly joined to the prostatic fascia. These findings suggest that to preserve the NVBs, the dissection plane should be posterior to the DVF, as the DVF is fused with the prostatic fascia; otherwise, the use of a dissection plane anterior to the DVF may damage the NVB (Fig. 5).

A previous study evaluating the relationship between the DVF and the MRF suggested that the optimal TME plane is anterior to the DVF. These authors failed to distinguish the DVF from the MRF, even at high microscopic magnification^11^. We believe that this discrepancy is due to technical limitations from the inevitable alterations of the local tissue during the processing of the specimens. To avoid this, we used micro-CT imaging, which does not alter the morphological relationship between the DVF, the MRF, and the NVBs.

To the best of our knowledge, this is the first study to use a nondestructive morphological method combined with histological observations of fresh cadaver tissue to identify the anatomic relationship between the DVF, the MRF, and the NVBs. Among several agents that can be used to enhance the contrast of the soft tissue, we used PTA as it produces high-contrast X-ray images in a wide variety of soft tissues^14^. Using a nondestructive morphological method, PTA staining can avoid altering the original configuration of the soft tissues during manual dissection as the fasciae are close to each other^21^.

Our study has some limitations. First, a relatively small number of specimens were evaluated, although this issue was unavoidable in light of the few cadavers available. Second, even though fresh cadavers were used, the specimens were collected from elderly cadavers. Fibrous tissues may have been altered due to degeneration of the prostate and seminal vesicles with age. Finally, data comparing the urogenital function in patients with rectal cancer in whom the dissection was performed anterior or posterior to the DVF are not provided. Therefore, a well-designed randomized controlled trial evaluating the optimal dissection plane for TME is needed.

Based on our micro-CT images and Masson’s trichrome staining results, the DVF appears as a multilayered structure that separates the rectum from the seminal vesicles, as well as the urogenital NVBs from the mesorectum at the posterior corner of the prostate. To preserve the PAN for urogenital function and optimal oncologic outcomes, the optimal dissection plane for TME in patients with rectal cancer should be located posterior to the DVF.

## Supplementary Information

Supplementary Video 1.

Supplementary Video 2.

Supplementary Video 3.
